# How skin anatomy influences transcutaneous bilirubin determinations: an in vitro evaluation

**DOI:** 10.1038/s41390-019-0471-z

**Published:** 2019-06-24

**Authors:** Marlijn D. van Erk, Alida J. Dam-Vervloet, Foky-Anna de Boer, Martijn F. Boomsma, Henrica van Straaten, Nienke Bosschaart

**Affiliations:** 1 Biomedical Photonic Imaging Group, Technical Medical Centre, University of Twente, Enschede, The Netherlands; 2 Medical Physics Department, Isala Hospital, Zwolle, The Netherlands; 3 Neonatology Department, Isala Hospital, Zwolle, The Netherlands; 4 Radiology Department, Isala Hospital, Zwolle, The Netherlands

## Abstract

**Background:**

Transcutaneous bilirubinometry is an effective screening method for neonatal hyperbilirubinemia. Current transcutaneous bilirubin (TcB) meters are designed for the “standard” situation of TcB determinations on the forehead or sternum of term newborns. We hypothesize that skin anatomy can considerably influence TcB determinations in non-standard situations—e.g., on preterm newborns or alternative body locations.

**Methods:**

A commercially available TcB meter (JM-105) was evaluated in vitro on phantoms that accurately mimic neonatal skin. We varied the mimicked cutaneous hemoglobin content (0–2.5 g/L), bone depth (0.26–5.26 mm), and skin maturity-related light scattering (1.36–2.27 mm^−1^) within the clinical range and investigated their influence on the TcB determination. To obtain a reference frame for bone depth at the forehead, magnetic resonance head scans of 46 newborns were evaluated.

**Results:**

The TcB meter adequately corrected for mimicked hemoglobin content. However, TcB determinations were influenced considerably by clinically realistic variations in mimicked bone depth and light scattering (deviations up to 72 µmol/L). This greatly exceeds the specified accuracy of the device (±25.5 µmol/L).

**Conclusion:**

As bone depth and light scattering vary with gestational maturity and body location, caretakers should be cautious when interpreting TcB measurements on premature newborns and non-standard body locations.

## Introduction

Since the introduction of the first transcutaneous bilirubin (TcB) meter in 1980,^1^ transcutaneous bilirubinometry has become an effective noninvasive method for screening hyperbilirubinemia in newborns and can be employed to reduce the number of invasive total serum bilirubin (TSB) measurements.^2–4^ Transcutaneous bilirubinometry cannot completely replace TSB determinations, since the TcB concentration is a physiologically different parameter from the TSB.^5^ Furthermore, it is known that TcB measurements correlate less well with the TSB for other body locations than the forehead and sternum^6^ and for premature newborns.^7,8^ Nevertheless, TcB measurements on premature newborns are becoming increasingly more common in clinical practice.^9^ Also measurements on other body locations than the forehead and the sternum are gaining popularity, e.g., to avoid the influence of ambient light^10^ or to investigate the cephalocaudal progression of jaundice.^11–13^

For both cases of TcB determinations on premature newborns and on non-standard body locations, the skin volume that is probed by the TcB meter may be intrinsically different from the standard situation (i.e., the forehead and sternum of term newborns) for which TcB meters have been designed and calibrated. In this article, we aim to provide a comprehensive understanding on which factors are important to consider and we quantitatively investigate their influence on the TcB determination. A better understanding of these factors is important for the interpretation of TcB determinations on patients, as well as the improvement of the clinical value of TcB meters in non-standard situations.

### TcB measurements and factors of influence

**Fig. 1:**
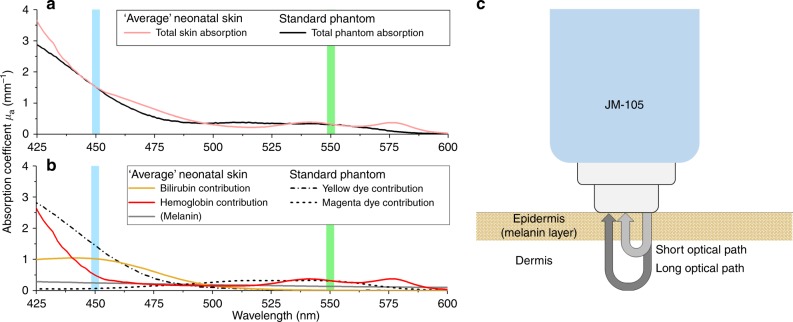
**a** Absorption spectra for average jaundiced neonatal skin and the standard phantom. At the evaluated wavelengths of 450 nm (blue) and 550 nm (green) by the transcutaneous bilirubin (TcB) meter, the absorption coefficients of the phantom are identical to those of the neonatal skin. **b** Individual contributions of bilirubin^26^ and hemoglobin^28^ to the total skin absorption, as well as the individual dye contributions to the total phantom absorption (obtained by transmission spectroscopy). The sum of the individual contributions yields the total skin, or phantom absorption, as depicted in **a**. Melanin absorption^28^ is shown for completeness but was not evaluated in this study. **c** Schematic overview of the working principle of the evaluated TcB meter (JM-105)

Commercially available TcB meters are optical devices: they measure bilirubin concentrations by evaluating light absorption around the bilirubin absorption peak at 450 nm (Fig. 1a, b), while correcting for the background absorption of hemoglobin and melanin. The TcB meter that was used in this study (the JM-105) evaluates hemoglobin absorption at a second wavelength of 550 nm (Fig. 1a, b) and corrects for epidermal melanin by measuring the difference in absorption at both wavelengths through both a short and a long optical path (Fig. 1c).^14^

Besides absorption, TcB meters rely heavily on the amount of light scattering by skin. Without light scattering, all non-absorbed photons would travel straight to the other side of the body and the TcB meter would not detect any signal. Therefore, the more the scattering, the smaller the probed skin volume. Light scattering by neonatal skin varies substantially between newborns^15^ and is influenced by gestational maturity, because concentrations of light-scattering collagen fibers increase with age.^16^

We further hypothesize that TcB measurements can be influenced by bone depth, which varies with skin thickness. In both standard measurement locations of TcB meters—the forehead and sternum—, the skin is directly supported by the bone. This can influence the TcB measurement in two different ways: (a) owing to the exerted pressure by the TcB meter on the skin, the underlying bone may support the temporal removal of blood from the probing volume, thereby facilitating the correction for hemoglobin absorption, and (b) in case of thin skin, the bone may become part of the probing volume, which will affect the amount of light that is scattered back into the TcB meter, due to a larger degree of backscattering by the bone compared to the skin.^17^

To summarize, the outcome of a TcB measurement relies on the optical absorption and scattering properties of the skin, as well as their spatial distribution within the probed skin volume—which is primarily determined by the factors mentioned above. We therefore hypothesize that the following factors influence the TcB determination: (1) hemoglobin concentration, (2) melanin concentration, (3) amount of light scattering by the skin, and (4) bone depth underneath the skin surface. Since several studies have shown that melanin concentrations influence the correlation between the TcB and the TSB,^3,18–20^ this factor was not investigated in this study.

### This study

We investigated the influence of hemoglobin concentration, the amount of light scattering by the skin, and bone depth on TcB measurements. Since literature is scarce on neonatal skin thickness, we evaluated forehead bone depth in premature and term newborns.

## Materials and methods

### Neonatal skin-mimicking phantoms

A systematic evaluation of the parameters of interest on TcB measurements in vivo is practically impossible, because none of them can be sufficiently controlled and/or quantified in patients. Therefore, we made use of aqueous neonatal skin phantoms that accurately mimic the average optical absorption and scattering properties of the neonatal skin, as well as the variation therein. These optical properties were derived from our previous work that involved an in vivo study on 60 neonates.^15^ Phantoms are generally used in medical imaging and sensing to evaluate the performance of a device. As shown in Fig. 1a, b, the total skin absorption by bilirubin and hemoglobin was mimicked by individual tuning of two dyes (Ecoline: Light-Yellow-201 and Magenta-337, Royal Thalens, The Netherlands). This results in a phantom absorption that is identical to that of neonatal skin at 450 and 550 nm (Fig. 1a). Light scattering was mimicked with varying concentrations of the standard tissue scattering phantom Intralipid (Intralipid^®^ 20%, Fresenius Kabi, Bad Homburg, Germany), using the work of Michels et al. to predict scattering as a function of concentration.^21^ We use the absorption coefficient *µ*_a_ and reduced scattering coefficient *µ*_s_’ to quantify the phantoms’ absorption and scattering properties, respectively, with units of inverse millimeters (mm^−1^).

Unless stated otherwise, our experiments were performed on a standard phantom that approximates the average optical properties of jaundiced neonatal skin at the sternum and forehead,^15^ with a TcB value of 136 µmol/L and a cutaneous hemoglobin concentration of 2.13 g/L. The corresponding optical properties are listed in Table 1 (standard phantom). For all phantoms, the TcB was measured as the average ± standard deviation (SD) of three measurements to obtain a good estimation of the spread in our data.

**Table 1:** Mimicked cutaneous parameters and corresponding phantom properties at 450 nm and 550 nm

Cutaneous parameter	Mimicked value	Corresponding *µ*_a_ (mm^−1^)		Corresponding *µ*_s_’ (mm^−1^)	
		450 nm	550 nm	450 nm	550 nm
*Standard phantom*					
TcB	136 µmol/L	1.54	0.35	2.00	1.63
Hemoglobin	2.13 g/L				
Reduced scattering	See last two columns				
Bone depth	5.26 mm				
*Hemoglobin series*					
TcB	136 µmol/L	1.02–1.64	0.01–0.38	2.00	1.63
Hemoglobin (11)^a^	0.00–2.50 g/L				
Reduced scattering	See last two columns				
Bone depth	5.26 mm				
*Scattering series*					
TcB (3)^a^	95–177 µmol/L	1.11–2.12	0.35	1.66–2.77	1.36–2.27
Hemoglobin	2.13 g/L				
Reduced scattering (5)^a^	See last two columns				
Bone depth	5.26 mm				
*Bone depth series*					
TcB (12)^a^	10–188 µmol/L	0.53–2.41	0.35	2.00	1.63
Hemoglobin	2.13 g/L				
Reduced scattering	See last two columns				
Bone depth (31)^a^	0.26–5.26 mm				

*TcB* transcutaneous bilirubin

^a^Marks the mimicked cutaneous parameter that was varied per phantom series (i.e., the independent variable), with the number of phantoms varied per series denoted between parentheses. For the scattering series, both reduced scattering (5 values) and TcB (3 values) were varied independently, resulting in a total number of 5 × 3 = 15 phantom measurements. For the bone depth series, both bone depth (31 values) and TcB (12 values) were varied independently, resulting in a total number of 31 × 12 = 372 phantom measurements

### Experimental set-up

All TcB measurements were performed with the JM-105 (serial number B3601086, Draeger Medical, Lübeck, Germany). The accuracy of the TcB measurements specified by the manufacturer is 25.5 µmol/L. To prevent damage, direct phantom contact was avoided by covering the measurement tip with a transparent, waterproof layer of a stretched Tegaderm^TM^ film dressing (1634W, 3M Healthcare, USA). Its thickness (44 ± 7 µm) was measured with optical coherence tomography, which is comparable to the neonatal epidermal thickness.^22^

Since the measurement tip of the JM-105 needs to be pressed down before a measurement can be obtained, the phantoms were covered by a rigid steel plate with an opening (*Ø* 8.8 mm) to accommodate the TcB meter (outer optical detection ring *Ø* 8.1 mm). Inside the opening, the plate thickness was 0.26 mm and direct contact between the Tegaderm covered measurement tip and the phantom was ensured. To minimize optical reflections, the plate was painted black and air bubbles that adhered to the phantom bottom were removed by gentile scraping with a rubber block. Air bubbles that adhered to the steel plate were effectively removed by making contact with the phantom bottom, prior to the measurement. For the investigation of the influence of bone depth, the distance between the measurement tip of the TcB meter and the bone mimicking layer was varied by translating the phantom with a manual stage with respect to the covering steel plate.

The reproducibility of the TcB determination with the Tegaderm-covered measurement tip in this measurement setting was excellent, with 0.77 µmol/L (SD of *N* = 180 measurements on one phantom with 6 different Tegaderm films, 30 measurements/film), accounting for ~0.6% of the measured TcB for the standard phantom.

### Influence of hemoglobin concentration

The influence of the hemoglobin concentration on the TcB measurement was evaluated by varying the mimicked cutaneous hemoglobin concentration in the standard phantom from 0.00 to 2.50 g/L in 11 steps (see Table 1: Hemoglobin series). Variations in cutaneous hemoglobin concentrations can be caused by variations in cutaneous vessel density, the hemoglobin concentration in blood, and the degree by which these cutaneous vessels are filled with blood. As we presume that blood is forced out of the skin during a TcB measurement, all but one phantom had a lower mimicked cutaneous hemoglobin concentration than the standard phantom.

### Influence of the light-scattering properties of the skin

The influence of the light-scattering properties of the skin on the TcB measurement was evaluated by varying the reduced scattering coefficient *µ*_s_’ at 550 nm from 1.36 to 2.27 mm^−1^ in 5 steps. This covers 90% of the variation in light scattering by the neonatal skin at the forehead and sternum.^15^ In a similar manner, the influence of light scattering was investigated for two series of phantoms with lower (95 µmol/L) and higher (177 µmol/L) mimicked bilirubin concentrations than the standard phantom (see Table 1: Scattering series).

### Influence of bone depth

Bone was mimicked with a diffusely scattering layer of white rubber at the bottom of the phantom. Taking the thickness of the phantom covering steel plate itself into account, the investigated range of mimicked bone depths was 0.26–5.26 mm in 31 steps. The influence of bone depth was investigated in 12 phantoms, with varying TcB values from 10 to 188 µmol/L (see Table 1: Bone depth series).

### Retrospective pilot evaluation of forehead bone depth in premature and term newborns

Knowledge on actual bone depth underneath the skin in patients is required to interpret our data on the influence of this factor. Therefore, we performed a retrospective pilot evaluation of forehead bone depth in the T1-weighted sagittal magnetic resonance (MR) head scans of 46 neonates (Table 2). These data were obtained between June 1, 2017 and June 1, 2018 at the Isala Hospital (Zwolle, The Netherlands) for medical reasons, other than this study. From all available neonatal MR head scans, MR scans were included based on image quality (i.e., absence of movement artifacts) and the availability of patient data (i.e., gender, birth weight, gestational age, and postnatal age). The distance between the skin surface and the skull was measured manually in Sectra (Sectra Ab, Sweden) at the forehead. The interobserver reproducibility of the measurement was evaluated as the SD of *N* = 60 measurements on 4 patients, with 15 measurements/patient. Ethical approval was obtained from an accredited medical research ethics committee (#1703017, MREC Zwolle, The Netherlands).

**Table 2:** Patient data for the retrospective pilot study on bone depth in *N* = 46 patients

	All patients (*N* = 46)		Female (*N* = 21)		Male (*N* = 25)	
	mean ± SD	range (min–max)	mean ± SD	range (min–max)	mean ± SD	range (min–max)
Birth weight (g)	2787 ± 1007	810–4870	2799 ± 1010	810–4400	2776 ± 1005	850–4870
Postmenstrual age (weeks)	40.4 ± 3.2	29.0–46.9	39.8 ± 3.7	29.0–45.7	40.9 ± 2.7	33.6–46.9
Gestational age (weeks)	36.4 ± 4.2	25.0–42.0	36.8 ± 4.3	28–42	36.1 ± 4.2	25–42
Postnatal age (days)	27.6 ± 30.7 (median: 6)	1–96	21.0 ± 28.4 (median: 6)	1–88	33.2 ± 31.3 (median: 12)	2–96
Bone depth (mm)	2.91 ± 0.77	1.10–4.60	2.84 ± 0.84	1.10–4.40	2.96 ± 0.70	1.60–4.60

## Results

### Influence of hemoglobin concentration

**Fig. 2:**
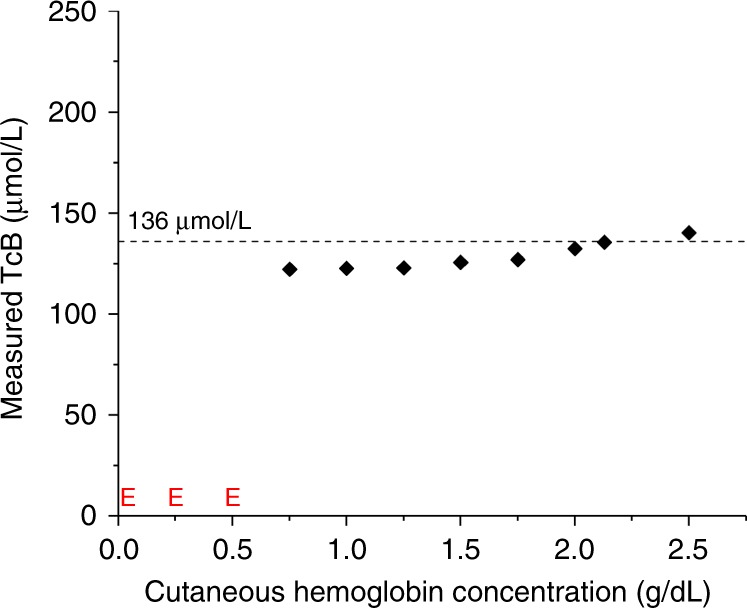
Measured transcutaneous bilirubin (TcB) as a function of the mimicked cutaneous hemoglobin concentration. All measurements were performed with a constant mimicked TcB value of 136 µmol/L, as indicated by the dashed line. Error bars fall behind the data points. E: device error (“abnormal measurement value”)

Figure 2 shows the measured TcB as a function of the mimicked concentration of cutaneous hemoglobin for phantoms with a constant mimicked TcB value of 136 µmol/L. For cutaneous hemoglobin concentrations >0.75 g/L, the measured TcB is relatively constant (129 ± 6.3 µmol/L). Although a moderate trend can be observed of increasing TcB with higher cutaneous hemoglobin concentration, this variation falls within the accuracy specified by the manufacturer (±25.5 µmol/L). For cutaneous hemoglobin concentrations <0.75 g/L, the TcB meter displays a measurement error (“abnormal measurement value”). We speculate that this is caused by the saturation of the device’s photodetector at 550 nm for very low absorption values.

### Influence of the light-scattering properties of the skin

**Fig. 3:**
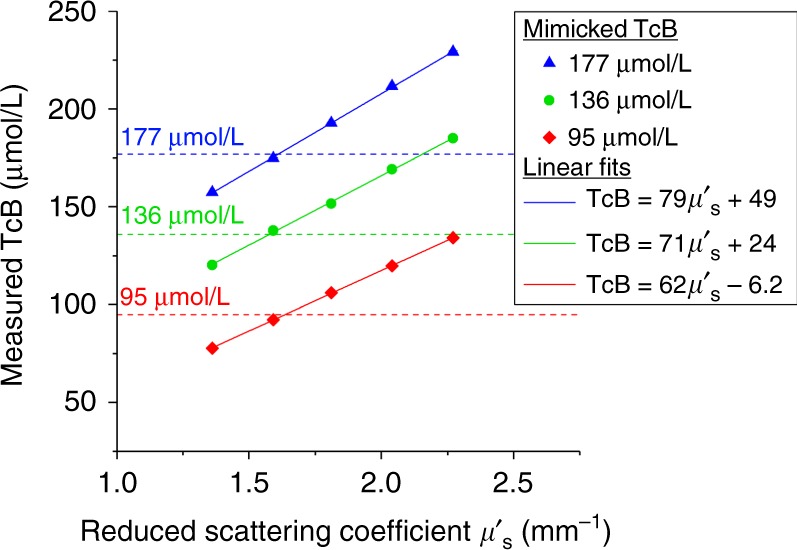
Measured transcutaneous bilirubin (TcB) as a function of the reduced scattering coefficient *µ*_s_’ at 550 nm. The three series represent phantoms with a constant mimicked TcB concentration (95, 136, and 177 µmol/L—indicated by the dashed lines), but with varying *µ*_s_’. Error bars fall behind the data points. All the linear fits through the data have a Pearson correlation coefficient *r* = 1.0

Figure 3 shows the measured TcB as a function of the reduced scattering coefficient *µ*_s_’ at 550 nm. The three different colors represent the three series of phantoms with varying *µ*_s_’ but with a fixed mimicked TcB concentration per series (red = 95 µmol/L, green = 136 µmol/L, blue = 177 µmol/L). If the TcB meter adequately corrects for the influence of skin scattering, the TcB would be constant at the expected TcB value as a function of *µ*_s_’ for each series of phantoms. However, we observe a large linear dependency of the TcB on *µ*_s_’, as quantified by the linear fits in the graph. The measured variation in the TcB amounts up to 72 µmol/L for the series with a mimicked TcB of 177 µmol/L. This greatly exceeds the accuracy specified by the manufacturer.

### Influence of bone depth

**Fig. 4:**
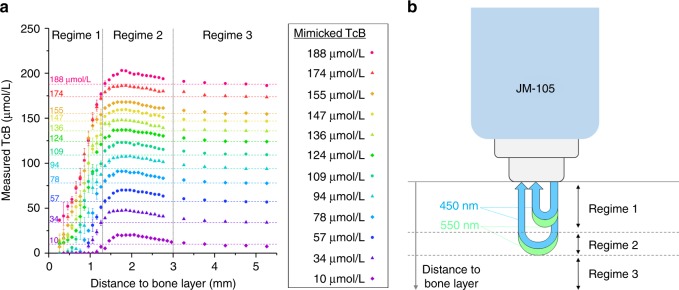
**a** Measured transcutaneous bilirubin (TcB) as a function of mimicked bone depth underneath the skin surface. The 12 series represent phantoms with each a constant mimicked TcB concentration (indicated by the horizontal dashed lines). Error bars may fall behind the data points. Roughly, the graph can be divided into three bone depth regimes, for which the boundaries of the position of the bone mimicking layer have been schematically depicted by the dashed lines in **b**. The blue and green arches illustrate the long and short optical paths of the blue (450 nm) and green light (550 nm), respectively

Figure 4a shows the measured TcB as a function of mimicked bone depth. The different colors represent 12 phantoms with a fixed mimicked TcB concentration (10–188 µmol/L). Roughly, we can divide the graph into three bone depth regimes, for which we briefly explain the observed results:

- Regime 1 (bone depth <1.3 mm). Both the long and the short optical path interfere with the mimicked bone layer (Fig. 4b). As a consequence, more light is detected than expected, which results in a lower apparent absorption, and thus an underestimation of the TcB.
- Regime 2 (1.3 mm ≤ bone depth ≤ 3.0 mm). The bone mimicking layer is deep enough to exclude any interference with the short optical path, as well as the blue wavelength (450 nm) in the long optical path. The green wavelength (550 nm) in the long optical path travels slightly deeper than the blue wavelength, due to a reduction in scattering for longer wavelengths.^23^ When this long, green optical path is the only path that interferes with the bone mimicking layer, the measured TcB value is corrected for a hemoglobin value that is lower than the actual hemoglobin absorption, resulting in an overestimation of the TcB value.
- Regime 3 (bone depth >3.0 mm). The bone mimicking layer is deep enough to exclude any interference with the long and the short optical path at both wavelengths (Fig. 4b). As a consequence, the measured TcB equals the expected TcB.

To summarize our results, only for bone depths >3.0 mm (regime 3), the measured TcB corresponds to the expected TcB (maximum deviation 5.3 µmol/L). For bone depths between 1.3 and 3.0 mm (regime 2), the TcB is overestimated up to 15.3 µmol/L above the expected value. This falls within the reported accuracy of the TcB meter (±25.5 µmol/L). For bone depths <1.3 mm (regime 1), the TcB is underestimated. It exceeds the accuracy specified by the manufacturer for bone depths <1.1 mm.

### Retrospective evaluation of forehead bone depth in premature and term newborns

**Fig. 5:**
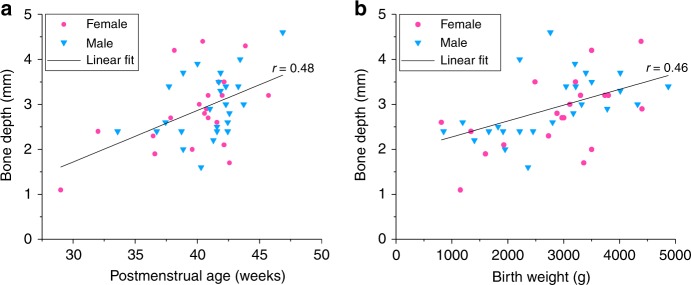
Patient data for the relation between bone depth underneath the skin surface at the forehead and **a** postmenstrual age (gestational+postnatal age) and **b** birth weight. The data points are individually marked for gender, but correlations were calculated for all data points together (*N* = 46)

Table 2 depicts the patient characteristics and data for the retrospective study on bone depth underneath the skin surface at the forehead of 46 patients. The measured bone depths varied between 1.10 and 4.60 mm. Figure 5 shows that moderate but significant correlations were found between bone depth and postmenstrual age (i.e., gestational+postnatal age, Pearson *r* = 0.48; *p* < 0.05), as well as bone depth and birth weight (Pearson *r* = 0.46; *p* < 0.05). Data were individually marked for gender, as bone depth may be related to gender-dependent postnatal growth. The interobserver reproducibility of the measurements was 0.18 mm.

## Discussion

The purpose of this study was to evaluate the influence of skin anatomy on the measured TcB. Hereto, we evaluated the JM-105 on neonatal skin phantoms in which we varied the mimicked hemoglobin concentration, light-scattering properties, and bone depth underneath the skin surface. All factors were varied within the range that naturally occurs in (premature) newborns. The accuracy of the TcB meter that has been specified by the manufacturer is 25.5 µmol/L. Below, we briefly summarize and discuss our findings.

### Influence of hemoglobin concentration

The TcB meter adequately corrects for the influence of hemoglobin absorption, as the measured TcB is only moderately affected by changes in hemoglobin concentration (SD ≤ 6.3 µmol/L). This variation falls within the specified accuracy by the manufacturer.

### Influence of the light-scattering properties of the skin

The measured TcB is influenced considerably by naturally occurring variations in light scattering by neonatal skin, with deviations amounting up to 72 µmol/L. This greatly exceeds the specified accuracy by the manufacturer, which may be explained by the fact that this specified accuracy was evaluated on a more homogeneous patient population in terms of gestational maturity. In our study, the light scattering was varied within 90% of the range that was encountered in neonates with a gestational maturity of 26–43 weeks.^15^ Even larger deviations may be expected for older neonates, as the collagen content-dependent light scattering by the skin further increases with gestational maturity.^16^ Future TcB meters may be improved by including one or more additional optical paths, as this allows to correct for the influence of light scattering.^24^

### Influence of bone depth

Only for bone depths >3.0 mm underneath the skin surface, the mimicked bone layer does not interfere with the TcB measurement. The TcB measurement is underestimated beyond the accuracy specified by the manufacturer for bone depths <1.1 mm, which coincides with the smallest bone depth at the forehead that was encountered in our retrospective MR study (1.10–4.60 mm). However, bone depth may be more shallow in the practice of a TcB measurement due to the exerted pressure on the skin by the TcB meter. Moreover, the limited number of patients in our MR study may underrepresent the full range of naturally occurring bone depths, and bone depths of 0.8 mm have been reported for other body locations.^25^ Although our phantom’s white rubber layer is only an approximation of an in vivo bone layer, our findings give an accurate estimation of the probing depth of the TcB meter. Any tissue structure that differs from skin tissue in optical absorption and scattering will therefore interfere with the TcB measurement when its depth is <3.0 mm underneath the skin surface. The amount of optical absorption—including the bilirubin concentration—and scattering will determine the severity of this interference, which can be quantified in follow-up phantom studies, or through Monte Carlo simulations. When in doubt, high-resolution ultrasound may assist in evaluating bone depth.^25^ Also, alternative optical methods such as spectroscopic optical coherence tomography may circumvent the influence of bone depth by evaluating bilirubin in a more confined probing volume.^26^

### Study limitations

This study involved a rigorous in vitro evaluation of the influence of skin anatomy on TcB measurements, since a systematic evaluation of the parameters of interest in vivo is currently impossible. Such a study would not only require a sufficient amount of variation in the parameters of interest, but also the technology to quantify them within the exact same volume as the TcB meter interrogates. In the near future, emerging technologies such as spectroscopic optical coherence tomography may be able to solve this issue.^26^

For this in vitro study, we made use of the neonatal skin-mimicking phantoms. At the detected wavelengths by the TcB meter (450 and 550 nm), these phantoms accurately mimic the average optical properties of the neonatal skin that have been previously assessed in vivo, as well as the variation therein.^15^ Variation in skin optical properties can be caused by variations in skin composition, which primarily involves skin maturity-related collagen content, bilirubin concentration, melanin concentration, and physiological variations in blood content. The latter not only affects optical absorption but is also known to affect the scattering properties of tissue.^27^ By systematically varying the phantom optical properties within the full range that has been encountered in vivo, we accounted for many of these physiological variations in this study. We considered water absorption to be negligible in this study, as the contribution of water to the total skin absorption is approximately a factor 10^4^–10^5^ lower than the contribution of hemoglobin at these wavelengths.^28^ We also assumed that the detected bandwidth at both wavelengths is <15 nm, to ensure a good resemblance of the skin absorption spectrum by the phantom dyes (Fig. 1a, b). Although the reproducibility of our measurements is high (0.77 µmol/L), our experimental set-up differs from the in vivo situation in the sense that we measured through a transparent Tegaderm film dressing and the TcB meter was supported by a thin plate. To some extent, the Tegaderm film dressing can be considered part of the skin-mimicking phantom, as it approaches the thickness of the neonatal epidermal layer.^22^ For the employed measurement geometry, we assume that the thin plate does not contribute to the detection of any significant optical reflections.

The skin-mimicking phantoms in this study are representative of Caucasian neonatal skin, because we did not mimic the epidermal melanin content. Although the influence of melanin content on TcB determinations has been evaluated previously in homogeneous phantoms,^20^ an interesting future direction will be to evaluate this factor in phantoms that incorporate an epidermal layer geometry. Future phantom studies will also allow to evaluate the reproducibility between different TcB meters in a controlled manner.

### Clinical implications

This study provides insight into the working mechanism of TcB meters and why TcB determinations correlate less well with TSB determinations for premature infants and non-standard body locations. This is important for the interpretation of TcB determinations on patients, as well as for the improvement of the clinical value of TcB meters in non-standard situations. Our findings demonstrate that TcB determinations are influenced considerably by clinically realistic variations in both the light-scattering properties of the skin, as well as bone depth underneath the skin surface. The specified accuracy of the TcB meter by the manufacturer is not valid for (1) shallow bone depths (≤1.1 mm), which are more likely to occur for premature, and low birth weight infants, and (2) natural variations in light scattering of the skin (≥0.5 mm^−1^) for both term and preterm infants. Owing to variations in optical illumination and detection geometry, other brands of TcB devices may perform differently.

In clinical practice, the accepted safety margins are larger than the accuracy specified by the manufacturer: when TcB values are <50 µmol/L below the phototherapy threshold, additional TSB determination is applied, and the decision to start treatment is always based on the TSB.^2^ Nevertheless, with deviations up to 72 µmol/L, our results demonstrate that also this safety margin can be exceeded. Moreover, this phantom study did not include the additional uncertainty that is introduced by the unknown extravasation ratio of bilirubin from blood to skin tissue and its physiological variation.^5^ Deviations may therefore be larger in clinical practice, and in particular TcB safety margins for premature infants need careful evaluation.

## Conclusion

Our most important findings are that TcB determinations in a neonatal skin-mimicking model are influenced considerably by clinically realistic variations in both the light-scattering properties of the skin and bone depth underneath the skin surface. Deviations up to 72 µmol/L were found, which greatly exceed the accuracy of the bilirubin meter as specified by the manufacturer (±25.5 µmol/L). As bone depth and light scattering by the skin are related to gestational maturity and body location, caretakers should be cautious when interpreting TcB measurements on premature newborns and non-standard body locations, i.e., other than the forehead and sternum.
